# Oscillayers: A dataset for the study of climatic oscillations over Plio‐Pleistocene time‐scales at high spatial‐temporal resolution

**DOI:** 10.1111/geb.12979

**Published:** 2019-07-22

**Authors:** Alexander Gamisch

**Affiliations:** ^1^ Department of Biosciences University of Salzburg Salzburg Austria

**Keywords:** anomaly, bioclim, ENM, interpolation, oscillations, palaeo‐climate, Pleistocene, Pliocene

## Abstract

**Motivation:**

In order to understand how species evolutionarily responded to Plio‐Pleistocene climate oscillations (e.g. in terms of speciation, extinction, migration and adaptation), it is first important to have a good understanding of those past climate changes per se. This, however, is currently limited due to the lack of global‐scale climatic datasets with high temporal resolution spanning the Plio‐Pleistocene. To fill this gap, I here present Oscillayers, a global‐scale and region‐specific bioclim dataset, facilitating the study of climatic oscillations during the last 5.4 million years at high spatial (2.5 arc‐minutes) and temporal (10 kyr time periods) resolution. This dataset builds upon interpolated anomalies (Δ layers) between bioclim layers of the present and the Last Glacial Maximum (LGM) that are scaled relative to the Plio‐Pleistocene global mean temperature curve, derived from benthic stable oxygen isotope ratios, to generate bioclim variables for 539 time periods. Evaluation of the scaled, interpolated estimates of palaeo‐climates generated for the Holocene, Last Interglacial and Pliocene showed good agreement with independent general circulation models (GCMs) for respective time periods in terms of pattern correlation and absolute differences. Oscillayers thus provides a new tool for studying spatial‐temporal patterns of evolutionary and ecological processes at high temporal and spatial resolution.

**Main types of variable contained:**

Nineteen bioclim variables for time periods throughout the Plio‐Pleistocene. Input data and R script to recreate all 19 bioclim variables.

**Spatial location and grain:**

Global at 2.5 arc‐minutes (4.65 x 4.65 = 21.62 km^2^ at the equator).

**Time period and grain:**

The last 5.4 million years. The grain is 10 kyr (= 539 time periods).

**Level of measurement:**

Data are for terrestrial climates (excluding Antarctica) taking sea level changes into account.

**Software format:**

All data are available as ASCII grid files.

## 1 INTRODUCTION

Understanding how species responded to past (e.g. Plio‐Pleistocene) climate oscillations is of great utility for understanding the evolution of organisms and their future response to anthropogenic climate change (Bálint et al., 2018; Comes & Kadereit, 1998; Espíndola et al., 2012; Haywood et al., 2009; Hewitt, 2000; Lawing & Polly, 2011; Myers, Stigall, & Lieberman, 2015). Spatially explicit palaeo‐climatic data have provided important insights into macroecology (Bálint et al., 2018; Couvreur et al., 2015; Kissling, Blach‐Overgaard, Zwaan, & Wagner, 2016; Rakotoarinivo et al., 2013), macroevolution (Meseguer et al., 2018), palaeobiology (Myers et al., 2015), systematics (Frajman et al., 2019; Younger et al., 2018), biogeography (Benítez‐Benítez, Escudero, Rodríguez‐Sánchez, Martín‐Bravo, & Jiménez‐Mejías, 2018; Espíndola et al., 2012; Silva, Antonelli, Lendel, Moraes, & Manfrin, 2018; Wang et al., 2015), palaeophylogeography (Lawing & Polly, 2011; Lawing, Polly, Hews, & Martins, 2016; Rödder et al., 2013) and conservation (Alsos, Alm, Normand, & Brochmann, 2009). However, such palaeo‐climatic data are currently restricted to a few time periods (e.g. 6 kyr, 21 kyr, 120 kyr and 3 Myr) (Braconnot et al., 2007; Haywood et al., 2011; Lawing & Polly, 2011; Lima‐Ribeiro et al., 2015; Otto‐Bliesner et al., 2006; but see Espíndola et al., 2012; Fordham et al., 2017; Singarayer & Valdes, 2010 for < 120 kyr time series and Brown, Hill, Dolan, Carnaval, & Haywood, 2018 for 787 kyr) not least because of their computationally expensive generation via global circulation models (GCMs; Lawing & Polly, 2011; Lima‐Ribeiro et al., 2015; Ramirez‐Villegas & Jarvis, 2010) that require further downscaling and calibration using interpolated observational data of current climates (e.g. WorldClim v. 1.4; Hijmans, Cameron, Parra, Jones, & Jarvis, 2005). Although these deep‐time climatic snapshots often serve as proxies for inferring the climatic dynamics of entire epochs (e.g. Benítez‐Benítez et al., 2018; Couvreur et al., 2015; Silva et al., 2018; Wang et al., 2015) they lack information on fine‐scaled climatic fluctuations through time as would be required for eco‐evolutionary studies (Espíndola et al., 2012; Fordham et al., 2017). Nevertheless, climate change between the present and the past (e.g. Last Glacial Maximum, LGM, *c.* 23–18 kyr BP; Peteet, 2018) has been used to interpolate climate trends in North America to a few earlier time periods (e.g. last 320 kyr, 4 kyr increments; last 23 Myr, 1 Myr increment; Lawing & Polly, 2011; Lawing et al., 2016; Rödder et al., 2013) by reference to the benthic stable oxygen isotope record (hereafter isotope record) (Collevatti, Terribile, Diniz‐Filho, & Lima‐Ribeiro, 2015). However, there is currently neither a global palaeo‐climatic dataset spanning the entire Plio‐Pleistocene at high spatial and temporal resolution nor a written procedure (protocol) detailing each step of the interpolation procedure.

**Figure 1:**
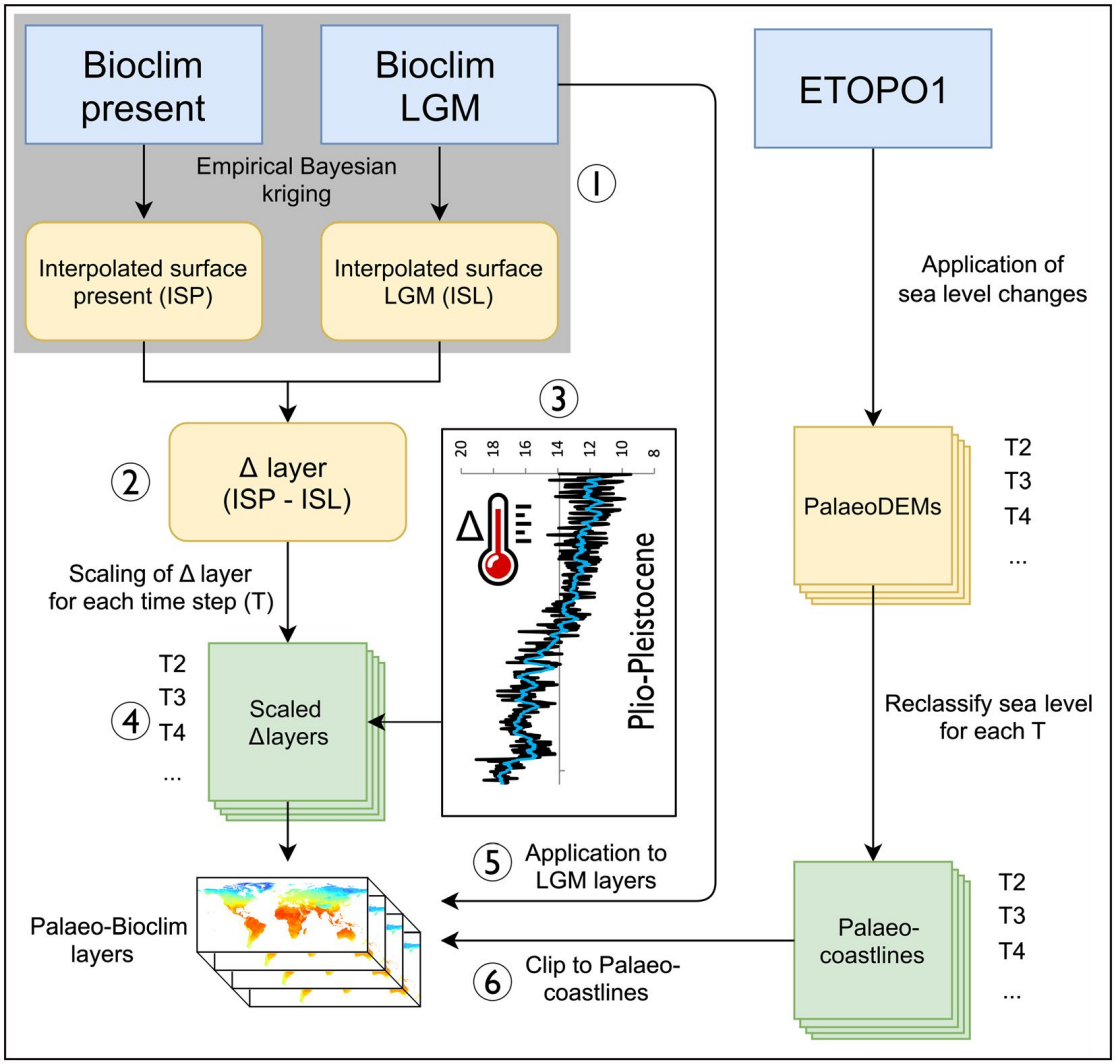
Flowchart for generating interpolated palaeo‐bioclim layers (Oscillayers). **Step 1**: Empirical Bayesian kriging interpolation of each variable for the present and the Last Glacial Maximum (LGM). **Step 2**: Computation of the Δ layers. **Step 3**: Calculation of surface temperature (Ts) differences between the LGM and proceeding time periods as derived from the isotope record for each time step (T). **Step 4**: Scaling the Δ layers relative to those differences of Step 3. **Step 5**: Application of the scaled Δ layers to the LGM variables for calibration. **Step 6**: Clipping each layer with the corresponding palaeo‐coastlines, as derived from reclassified digital elevation models (DEMs), to obtain the calibrated palaeo‐bioclim layers of each time period (Oscillayers) [Colour figure can be viewed at https://www.wileyonlinelibrary.com]

To fill this gap, I here present Oscillayers, a ready to use global terrestrial palaeo‐climatic dataset for all 19 conventional bioclim variables (Hijmans et al., 2005), spanning continuously from the beginning of the Pliocene (5.4 Myr) to the LGM (*c.* 20 kyr BP) in steps of 10 kyr plus input data (Δ layers) and an R script, to recreate those variables for the respective time periods (Figure 1). All scaled and interpolated 19 palaeo‐bioclim variables were also evaluated against independent GCMs for three time periods [i.e. Holocene Climate Optimum (HOL), *c.* 6 kyr BP (http://www.worldclim.org/paleo-climate1; Hijmans et al., 2005); Last Interglacial (LIG), *c.* 120 kyr BP (http://www.worldclim.org/paleo-climate1; Otto‐Bliesner et al., 2006); mid‐Pliocene Warm Period (PLIO), *c.* 3.3 to 3.0 Myr BP (http://ecoclimate.org/; Lima‐Ribeiro et al., 2015)] in terms of pattern correlations and absolute differences. Oscillayers thus provides a novel tool for studying climatic fluctuations spanning the Plio‐Pleistocene at high temporal and spatial resolution. Potential applications for eco‐evolutionary studies are briefly discussed.

## 2 METHODS

### 2.1 Data generation

Nineteen bioclim variables (Bio1–Bio19) were obtained from WorldClim v. 1.4 (Hijmans et al., 2005) for current (*c.* 1960–2000) and LGM (Community Climate System Model, CCSM4; Gent et al., 2011) conditions at 2.5 arc‐minutes resolution to generate palaeo‐bioclim variables for each time period (T) using the following multistep procedure (Figure 1; see also Supporting Information Appendix S1 for technical details). **Step 1**: Empirical Bayesian kriging (EBK; Krivoruchko, 2012) was used to calculate geographically interpolated surfaces of each variable for the present (interpolated surface present; ISP) and the LGM (interpolated surface LGM; ISL). This step provides baseline climate estimates for areas that are now submerged but were subaerial during past glacial cycles when sea levels were lower under the assumption of spatial correlation between coastal and off coastal climates. **Step 2:** Δ layers, representing the climate change during the LGM [i.e. when the average global temperature (9.46 °C) was 4.44 °C colder than today; Hansen, Sato, Russell, and Kharecha (2013)], were then computed between the ISP and ISL (e.g. Bio1_Δ_ = Bio1_ISP_ – Bio1_ISL_; see also Figure S1 in Supporting Information Appendix S1). **Step 3:** Differences in surface temperature (Ts) between the LGM (*c.* 20 kyr) and those of the last 5.4 Myr in time steps of 10 kyr (= 539 steps), as derived from the isotope record (Hansen et al., 2013), were calculated (see Table S1 in Supporting Information Appendix S1). **Step 4:** The Δ layers were then scaled relative to those differences (cf. Lawing & Polly, 2011; Rödder et al., 2013) in relation to the temperature differences between the present and the LGM [e.g. Bio1_ΔT_ = Bio1_Δ_ * (Ts_T_ – Ts_LGM_)/(Ts_present_ – Ts_LGM_)]. These scaled and interpolated Δ layers thus represent the climate change between the LGM and the preceding time periods (e.g. T3 = 30 yrs, T4 = 40 kyr, …). **Step 5:** In a further step, the interpolated Δ layers were applied to the LGM layers to generate calibrated palaeo‐bioclim layers of each time period (e.g. Bio1_LGM_ + Bio1_ΔT_ = Bio1_T_) as inspired by the Delta method (Ramirez‐Villegas & Jarvis, 2010; see also http://www.worldclim.org/downscaling). This implies that the underlying spatial pattern of each modelled time period is driven by the scaled differences between the LGM and the present. **Step 6:** Finally, to obtain terrestrial layers with palaeo‐coastlines for each time period, each layer was clipped using land masks derived from the resampled ETOPO1 Global Relief Model (Amante & Eakins, 2009; https://doi.org/10.7289/V5C8276M) after applying corresponding changes in eustatic sea level (Hansen et al., 2013; see Table S1 in Supporting Information Appendix S1) via reclassification (Willmes, Becker, Brocks, Hütt, & Bareth, 2017). All steps were done using arcgis v.10.4 (ESRI, Redland, CA) and the R package “raster” v. 2.6–7 (Hijmans & van Etten, 2017).

### 2.2 Data validation

The ability of Oscillayers to reproduce independent data (skill and validity; see Fordham et al., 2017 and references therein) was evaluated by testing whether it can reproduce modelled past climates (HOL, LIG and PLIO) in more or less the same way as two highly correlated and commonly employed GCMs (i.e. CCSM4: Gent et al., 2011; Model for Interdisciplinary Research on Climate ‐ Earth System Model, MIROC‐ESM: Watanabe et al., 2011) of the recent past (see Lawing & Polly, 2011; Rödder et al., 2013; Varela, Lima‐Ribeiro, & Terribile, 2015) (see Figure S2 in Supporting Information Appendix S1 for the validation procedure). Although inter‐model comparisons are not possible for time periods (e.g. LIG, PLIO) that are represented by only a single GCM (Lima‐Ribeiro et al., 2015; Otto‐Bliesner et al., 2006), interpolation performances can nonetheless still be compared with more recent time periods in such cases (see Lawing & Polly, 2011).

Bioclim variables of three time periods used for validation (i.e. HOL_CCSM_, LIG, PLIO_CCSM_) and of two time periods used for evaluation (HOL_CCSM_, HOL_MIROC_, LGM_CCSM_, LGM_MIROC_) were obtained from WorldClim (HOL, LGM, LIG) and ecoClimate (PLIO; Lima‐Ribeiro et al., 2015). PLIO_CCSM_ bioclim variables were downscaled from 30 arc‐minutes resolution to 2.5 arc‐minutes resolution via the Delta method using the modern (1950–1999) CCSM model as baseline. The bioclim variables of three time periods (CCSM_HOL_, LIG, CCSM_PLIO_) were compared with the Oscillayers generated for respective periods in terms of pattern correlation and absolute difference (see below). Results were then compared (in similar terms) with inter‐model comparisons between the GCMs for HOL and LGM, that is CCSM and MIROC, respectively (see also Lawing & Polly, 2011; Rödder et al., 2013). Pairwise correlations were calculated in sdmtoolbox v. 2.2b (Brown, Bennett, & French, 2017), using Pearson's correlation coefficient (*r*), a commonly used metric for evaluating the skill of modelled climatic variables (Fordham et al., 2017). This coefficient can range between +1 and −1, indicating a positive or negative relationship, respectively, while a coefficient of 0 indicates that two layers are independent from each other (Brown et al., 2017).

## 3 RESULTS

### 3.1 Data validation

**Figure 2:**
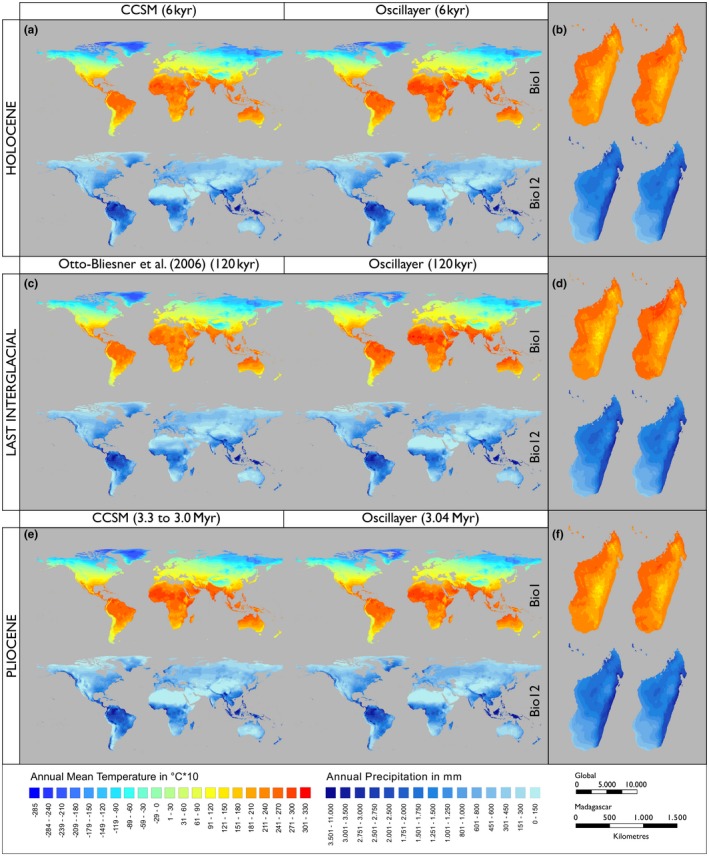
Oscillayers show good agreement with independent global circulation models (GCMs). Comparison of GCM‐derived and interpolated layers (Oscillayers) for annual mean temperature (Bio1) and annual precipitation (Bio12) at global and regional (Madagascar; see also Figure S3 in Supporting Information Appendix S1) scales for the Holocene (HOL: a, b), Last Interglacial (LIG: c, d) and Pliocene (PLIO: e, f) [Colour figure can be viewed at https://www.wileyonlinelibrary.com]

For the 19 bioclim variables, results showed generally good agreement between the interpolated palaeo‐bioclim layers (Oscillayers) of the three validation time periods (HOL, LIG, PLIO) and the corresponding independent GCMs (see Figure 2; see also Figure S3 in Supporting Information Appendix S1). Especially for Bio1 and Bio12 the interpolated variables were highly correlated with independent GCMs of the respective periods (HOL: *r*_Bio1_ = .999, *r*_Bio12_ = .993; LIG: *r*_Bio1_ = .970, *r*_Bio12_ = .962; PLIO: *r*_Bio1_ = .957, *r*_Bio12_ = .935; see also Table 1). The same was true for the remaining 17 bioclim variables (mean *r*_HOL_ = .978, range: .906–1.0; *r*_LIG_ = .877, .597–.975; *r*_PLIO_ = .904, .684–.963). The inter‐model pattern correlations between CCSM and MIROC for the HOL and LGM, respectively, were generally smaller than those of Oscillayers‐HOL_CCSM_ but tendentially higher than Oscillayers‐LIG and Oscillayers‐PLIO (Table 1).

**Table 1:** Pearson correlation coefficients (*r*) and absolute differences (2.5–97.5% quantiles) between scaled, interpolated palaeo‐bioclim variables (Oscillayers) generated for the Holocene (HOL), Last Interglacial (LIG) and Pliocene (PLIO) and independent general circulation models (GCMs) for respective time periods and between Community Climate System Model (CCSM) and Model for Interdisciplinary Research on Climate (MIROC) models for both HOL and the Last Glacial Maximum (LGM)

Bioclim variable	Pearson's correlation coefficient (*r*)					Absolute differences (2.5–97.5% quantiles)				
	Oscillayer–HOL_CCSM_	Oscillayer–LIG	Oscillayer–PLIO_CCSM_	HOL_CCSM_–HOL_MIROC_	LGM_CCSM_–LGM_MIROC_	Oscillayer–HOL_CCSM_	Oscillayer –LIG	Oscillayer–PLIO_CCSM_	HOL_CCSM_–HOL_MIROC_	LGM_CCSM_–LGM_MIROC_
Bio1 = annual mean temperature	.999^b^	.970	.957	.998	.989	3−23^b^	5−60^a^	1−48^a^	0–26	1–89
Bio2 = mean diurnal range	.991^b^	.829^a^	.926^a^	.968	.752	0−11^b^	0−38^a^	0−21^b^	0–23	0–63
Bio3 = isothermality	.992^a^	.921	.684	.992	.977	0−9^a^	0–22	1–54	0–7	0–12
Bio4 = temp. seasonality	.998^a^	.963^a^	.957	.998	.960	19−1489^a^	250−4969^a^	16−3128^a^	10–994	23–5193
Bio5 = max. temp. of warmest month	.993^b^	.899	.903	.988	.962	1−25^b^	1−85^a^	5–148	0–42	1–117
Bio6 = min. temp. of coldest month	.999^b^	.969	.962	.999	.982	6−36^a^	2−145^a^	5−117^a^	0–24	1–161
Bio7 = temp. annual range	.997^b^	.939^a^	.947^a^	.995	.931	1−49^a^	4–201	1−102^a^	0–40	1–174
Bio8 = mean temp. of wettest quarter	.972^b^	.803	.817	.954	.941	0−71^b^	1−162^a^	6–171	0–77	1–165
Bio9 = mean temp. of driest quarter	.992^b^	.939	.942	.990	.971	1−83^b^	1−193^a^	5–218	0–86	1–198
Bio10 = mean temp. of warmest quarter	.995^b^	.924	.913	.993	.975	0−22^b^	1−52^a^	7–130	0–28	1–95
Bio11 = mean temp. of coldest quarter	1.000^b^	.975	.963	.999	.988	5−31^a^	2−98^a^	6−112^a^	0–26	1–141
Bio12 = annual precipitation	.993^b^	.962^a^	.935	.982	.952	1−247^b^	3−424^b^	3−479^a^	1–426	1–643
Bio13 = prec. of wettest month	.968^b^	.880	.928	.949	.929	0−76^a^	3–449	0−83^b^	0–145	0–134
Bio14 = prec. of driest month	.978^b^	.907	.910	.970	.911	0−18^a^	0–34	0−28^a^	0–18	0–32
Bio15 = prec. seasonality	.961^a^	.760	.942^a^	.978	.906	0−21^a^	1–80	0−24^a^	0–20	0–34
Bio16 = prec. of wettest quarter	.983^b^	.924	.933	.964	.942	0−188^b^	1–323	1−212^b^	0–301	0–310
Bio17 = prec. of driest quarter	.983^b^	.932^a^	.907	.980	.922	0−54^b^	0−93^a^	0−92^a^	0–55	0–103
Bio18 = prec. of warmest quarter	.913^a^	.746	.902^a^	.936	.896	0−248^a^	1–389	1−206^b^	0–215	0–319
Bio19 = prec. of coldest quarter	.906^a^	.597	.841^a^	.915	.836	0−208^a^	0–616	0−264^a^	0–173	0–307

prec. = precipitation.

a Indicates that the Oscillayers‐GCM comparisons are more correlated (left in Table) or have lower absolute differences (right in Table) than the LGM_CCSM_‐LGM_MIROC_ inter‐model comparisons, respectively. Absolute differences are in the units of the respective bioclim variable.

b Indicates that the Oscillayers‐GCM comparisons are more correlated (left in Table) or have lower absolute differences (right in Table) than the HOL_CCSM_‐HOL_MIROC_, respectively.

Absolute differences in the 19 bioclim variables between the Oscillayers and the independent GCMs (for HOL, LIG and PLIO) compared favourably with those derived between the CCSM and MIROC variables for the HOL and LGM when judged by the 2.5–97.5% quantiles. In detail, Oscillayers‐HOL_CCSM_ absolute differences were smaller compared to those of HOL_CCSM_‐HOL_MIROC_ and LGM_CCSM_‐LGM_MIROC_ for nine and 19 variables, respectively (Table 1). Similarly, Oscillayers‐LIG and Oscillayers‐PLIO differences were smaller compared to those of LGM_CCSM_‐LGM_MIROC_ for 11 and 14 variables, respectively (Table 1). Overall, the generated palaeo‐bioclim layers showed good agreement with independent GCMs (HOL, LIG and PLIO), with differences being mostly smaller than those between the commonly used CCSM and MIROC models for the HOL and LGM, respectively. Hence, the current approach provides a sufficiently robust approximation of palaeo‐climate conditions throughout the Plio‐Pleistocene.

## 4 DISCUSSION

### 4.1 Applications

Oscillayers provides climatic data for 19 bioclim variables (see Supporting Information Appendices S2 and S3 for representative animations through time for Bio1 and Bio12), plus input data (see Table S2 in Supporting Information Appendix S1) and an R script, to recreate those variables for time periods spanning the early Pliocene (5.4 Myr) to the LGM (*c.* 20 kyr) in steps of 10 kyr.

Oscillayers can be used for testing a variety of eco‐evolutionary hypotheses over this time period, for example, about climate‐induced range changes of taxa or climate‐related patterns of diversification (speciation, extinction) and adaptation. This can be facilitated by projecting ecological niche models (ENMs) of ecosystems or species (extant or extinct) onto these palaeo‐climatic layers (e.g. Espíndola et al., 2012; Roberts & Hamann, 2012), or by reconstructing ancestral climatic envelopes along molecular phylogenies (Lawing & Polly, 2011; Lawing et al., 2016; Meseguer et al., 2018; Rödder et al., 2013; Yesson & Culham, 2006). Such spatially explicit models through time, either derived from ENMs (assuming niche conservatism) or ancestral climatic envelope reconstructions (taking niche divergence into account) might also be used for phylogeographic inferences “as is”, or for modelling and testing population demographic hypotheses within a coalescent framework (Collevatti et al., 2015, 2013). Other potential applications include, for example, the generation of PalaeoENMs via georeferenced fossils (Myers et al., 2015); the testing of biodiversity‐related hypotheses about palaeo‐climatic stability in the tropics (e.g. Couvreur et al., 2015; Kissling et al., 2016; Rakotoarinivo et al., 2013); the testing of predictions of the glacial‐sensitive model of island biogeography (Fernández‐Palacios et al., 2016; Norder et al., 2019) or the facilitation of landscape connectivity (dispersal corridor) analyses over time in a conservation context (Eberle, Rödder, Beckett, & Ahrens, 2017; Yu et al., 2015).

### 4.2 Limitations and caveats

The Oscillayers framework presented herein assumes that past climates can be described by relative differences between modern and Quaternary climates as guided by the isotope record (Collevatti et al., 2015; Lawing & Polly, 2011; Rödder et al., 2013). As a corollary, uncertainty of the interpolations may increase with time when this assumption becomes less likely to hold. Also, as the Oscillayers framework broadly assumes a modern continental configuration it cannot explicitly account for spatial effects of large‐scale geological events (e.g. the Messinian Salinity Crisis, *c.* 5.96 to 5.33 Myr). The current approach is therefore unlikely to be easily extended into pre‐Pliocene time periods. Also, the underlying GCMs used for the generation, validation and evaluation of Oscillayers are fraught with uncertainty, too (e.g. downscaling artefacts, parameters and functions used; Hargreaves, 2010; Lima‐Ribeiro et al., 2015; Varela et al., 2015; Wiens, Stralberg, Jongsomjit, Howell, & Snyder, 2009). Hence, the palaeo‐climates derived should be cross‐validated using fossil and/or molecular evidence (Alsos et al., 2009; Collevatti et al., 2013; Espíndola et al., 2012; Roberts & Hamann, 2012). Finally, a non‐trivial task is the generation of highly resolved palaeo‐coastlines through time (e.g. Norder et al., 2018, and references therein). Here, this was accomplished by using simple land masks for the palaeo‐coastlines of each of the 539 time periods based on reclassified current bathymetry and topography (cf. Willmes et al., 2017), albeit without taking regional topographic peculiarities on a global scale for each time period into account. Consequently, for technical reasons, land masks treat landlocked areas below sea level (e.g. the Qattara Depression) as missing data. Nevertheless, users are free to refine or regenerate the present land masks for their study region of interest (see also the Data Accessibility section).

## BIOSKETCH

**Alexander Gamisch** is a post‐doctoral researcher at the University of Salzburg. He is an evolutionary biologist interested in macroevolution, macroecology and biogeography. He is investigating these subjects on a broad scale, especially in tropical *Bulbophyllum* orchids, but also in plant and animal taxa from temperate Europe.

## Data Availability

The dataset (the 19 bioclim variables for 539 time periods throughout the Plio‐Pleistocene as well as the Δ layers and land masks to recreate variables) and the corresponding R script are available at the Dryad digital repository [doi: https://doi.org/10.5061/dryad.27f8s90]. The 19 bioclim variables and Δ layers are available as ASCII (ESRI) text files (see also Supporting Information Appendices S2 and S3 for representative animations through time for Bio1 and Bio12). Instead of computing the variables at a global scale for all time periods the user may choose to limit the calculations to the study region and or subsets of time periods of interest prior to running the script in order to save time and disk space. For this purpose shapefiles covering major geographic regions (e.g. Europe, Africa, North America, etc.) are provided but users are also free to tailor them to their own custom study region.
