# Supplementary material for: Oscillayers: A dataset for the study of climatic oscillations over Plio‐Pleistocene time‐scales at high spatial‐temporal resolution
Source: Glob Ecol Biogeogr. 2019 Jul 22;28(11):1552–60. doi: 10.1111/geb.12979 (PMC6853231; doi:10.1111/geb.12979)
Supplement: Supplementary file 1 [file GEB-28-1552-s001.docx]

**Supporting Appendix 1**

**Oscillayers: A data set for the study of climatic oscillations over Plio-Pleistocene time scales at high spatial-temporal resolution**

**INCLUDING:** Supplementary Methods, Tables S1-S2, Figure S1-S3

**Supplementary Methods**

**Data Generation**

**Step 1:** Bioclim variables of the present and LGM were converted to point features using the *raster to point* function of ArcGIS. This step converts each raster cell into point features, positioned at the centre of the cell they represent, with the original raster value (e.g. temperature) as attribute. These point features were then used to create a continuous interpolated surface via Empirical Bayesian Kriging (EBK). EBK is a sophisticated probabilistic interpolation method that uses the differences between point measures (= semivariance) to summarize the spatial relationships in variables (Krivoruchko, 2012). Unlike traditional Kriging methods, EBK can accurately interpolate nonstationary data and accounts for potential errors in estimating the underlying semivariogram (= function of distance and direction separating two locations) through repeated simulations (Krivoruchko, 2012; Caprarelli & Fletcher, 2014). Like all spatial prediction methods, EBK assumes some level of spatial auto-correlation in that, geographically distant values are less similar than closer ones (Krivoruchko, 2012; Caprarelli & Fletcher, 2014). EBK was performed for each of the 19 bioclim variables of the present and the LGM using the `Geostatistical Analyst Wizard´ extension as implemented in ArcGIS. The resulting geostatistical surfaces were then converted to raster via the *GA layer to grid* command using the original resolution of the input (2.5 arc minutes) as output cell size to generate the interpolated surfaces for the present (ISP) and the LGM (ISL). **Step 2:** For each of the 19 bioclim variables, differences between the ISP and ISL were computed using the ArcGIS *raster calculator* tool and subsequently clipped to LGM coastlines (see also Step 6) to generate the Δ layers (see Figure S1). **Steps 4** & **5** were performed with the *calc* and *arith-methods* functions of the R package `raster´ v. 2.6-7 (Hijmans & van Etten, 2017). Negative values were converted to zero for the precipitation variables Bio12–Bio19 (cf. Wagner et al., 2017), using the *reclassify* function of the same program. All variables were rounded to integers to safe disk space. **Step 6**: The ETOPO1 Global Relief Model (Amante & Eakins, 2009; doi:10.7289/V5C8276M) was resampled to 2.5 arc-minutes resolution through a nearest neighbor assignment procedure and copied to the extent of the Δ layers, using the *resample* and *copy raster* functions of ArcGis, respectively. The resulting digital elevation model (DEM) was then further modified by adding changes in sea-level (Hansen et al., 2013; see Table S1 in Supporting Appendix 1) for each time period (T), using the *calc* function. For each T, areas between 0 and -15,000 m below sea level were reclassified as water (no data) and land areas > 0 m were reclassified to 1, using the *reclassify* function. Based on the resulting binary land masks (for each T), and using the *mask* function of `raster´, each calibrated palaeo-bioclim layer (of each respective T) was clipped to obtain the final Oscillayers with corrected palaeo-coastlines.

**Supplementary References**

Caprarelli, G., & Fletcher, S. (2014). A brief review of spatial analysis concepts and tools used for mapping, containment and risk modelling of infectious diseases and other illnesses. *Parasitology*, 141, 581–601.

Wagner, M., Trutschnig, W., Bathke, A. C., & Ruprecht, U. (2018). A first approach to calculate BIOCLIM variables and climate zones for Antarctica. *Theoretical and Applied Climatology*, 131, 1397–1415.

**Supporting Tables**

**Table S1 Surface mean temperature (Ts) and sea-level changes (SL) compared to the Late Holocene level as estimated by Hansen et al. (2013) based on oxygen isotope records of ocean sediments.** First column shows age of the ocean sediment *sensu* Hansen et al. (2013) that was taken as representative for a given time period (T) for the purpose of the present study. Scaling factor used for scaling of the Δ layers.

| **Myr before present (Hansen et al. 2013)** | **Time period (T) in 10kyr** | **Ts (°C)** | **SL (m)** | **Δ LGM Ts** | **Scaling factor** |
| --- | --- | --- | --- | --- | --- |
| 0.02 | 2 | 9.46 | -118.8 | 0 | 0 |
| 0.03 | 3 | 10.57 | -91.2 | 1.11 | 0.250 |
| 0.04 | 4 | 10.84 | -84.7 | 1.38 | 0.311 |
| 0.05 | 5 | 10.67 | -88.9 | 1.21 | 0.273 |
| 0.06 | 6 | 11 | -80.6 | 1.54 | 0.347 |
| 0.069 | 7 | 11.35 | -72 | 1.89 | 0.426 |
| 0.08 | 8 | 12.74 | -37.5 | 3.28 | 0.739 |
| 0.09 | 9 | 12.2 | -50.9 | 2.74 | 0.617 |
| 0.1 | 10 | 12.74 | -37.4 | 3.28 | 0.739 |
| 0.109 | 11 | 12.41 | -45.7 | 2.95 | 0.664 |
| 0.12 | 12 | 14.52 | 2 | 5.06 | 1.140 |
| 0.131 | 13 | 11.83 | -60.1 | 2.37 | 0.534 |
| 0.14 | 14 | 9.97 | -106.2 | 0.51 | 0.115 |
| 0.151 | 15 | 10.13 | -102.1 | 0.67 | 0.151 |
| 0.16 | 16 | 10.37 | -96.3 | 0.91 | 0.205 |
| 0.171 | 17 | 11.27 | -73.9 | 1.81 | 0.408 |
| 0.18 | 18 | 11.05 | -79.4 | 1.59 | 0.358 |
| 0.19 | 19 | 12.71 | -38.3 | 3.25 | 0.732 |
| 0.2 | 20 | 13.52 | -18 | 4.06 | 0.914 |
| 0.2115 | 21 | 13.23 | -25.3 | 3.77 | 0.849 |
| 0.22 | 22 | 12.85 | -34.8 | 3.39 | 0.764 |
| 0.229 | 23 | 12.29 | -48.6 | 2.83 | 0.637 |
| 0.24 | 24 | 13.56 | -17 | 4.1 | 0.923 |
| 0.2493 | 25 | 10.57 | -91.3 | 1.11 | 0.250 |
| 0.26 | 26 | 11.15 | -76.8 | 1.69 | 0.381 |
| 0.27 | 27 | 10.75 | -86.8 | 1.29 | 0.291 |
| 0.28 | 28 | 12.27 | -49.2 | 2.81 | 0.633 |
| 0.29 | 29 | 12.31 | -48.2 | 2.85 | 0.642 |
| 0.3 | 30 | 11.35 | -71.8 | 1.89 | 0.426 |
| 0.3105 | 31 | 13.09 | -28.6 | 3.63 | 0.818 |
| 0.32 | 32 | 13.33 | -22.8 | 3.87 | 0.872 |
| 0.3292 | 33 | 13.86 | -9.6 | 4.4 | 0.991 |
| 0.34 | 34 | 11.66 | -64.2 | 2.2 | 0.495 |
| 0.3503 | 35 | 10.54 | -92.1 | 1.08 | 0.243 |
| 0.36 | 36 | 11.3 | -73.2 | 1.84 | 0.414 |
| 0.3716 | 37 | 11.56 | -66.8 | 2.1 | 0.473 |
| 0.38 | 38 | 11.93 | -57.5 | 2.47 | 0.556 |
| 0.3898 | 39 | 12.82 | -35.5 | 3.36 | 0.757 |
| 0.4 | 40 | 14.15 | -2.5 | 4.69 | 1.056 |
| 0.41 | 41 | 13.59 | -16.3 | 4.13 | 0.930 |
| 0.42 | 42 | 11.87 | -59 | 2.41 | 0.543 |
| 0.43 | 43 | 10.22 | -99.9 | 0.76 | 0.171 |
| 0.44 | 44 | 9.83 | -109.7 | 0.37 | 0.083 |
| 0.45 | 45 | 10.17 | -101.1 | 0.71 | 0.160 |
| 0.46 | 46 | 10.82 | -85.1 | 1.36 | 0.306 |
| 0.471 | 47 | 10.64 | -89.6 | 1.18 | 0.266 |
| 0.48 | 48 | 11.65 | -64.6 | 2.19 | 0.493 |
| 0.49 | 49 | 13.23 | -25.2 | 3.77 | 0.849 |
| 0.5 | 50 | 12.42 | -45.4 | 2.96 | 0.667 |
| 0.511 | 51 | 12.01 | -55.6 | 2.55 | 0.574 |
| 0.52 | 52 | 11.7 | -63.1 | 2.24 | 0.505 |
| 0.53 | 53 | 11.73 | -62.5 | 2.27 | 0.511 |
| 0.54 | 54 | 11.4 | -70.6 | 1.94 | 0.437 |
| 0.55 | 55 | 10.61 | -90.3 | 1.15 | 0.259 |
| 0.56 | 56 | 11.76 | -61.8 | 2.3 | 0.518 |
| 0.5707 | 57 | 12.72 | -38 | 3.26 | 0.734 |
| 0.58 | 58 | 12.94 | -32.6 | 3.48 | 0.784 |
| 0.5898 | 59 | 12.71 | -38.1 | 3.25 | 0.732 |
| 0.6 | 60 | 12.68 | -39 | 3.22 | 0.725 |
| 0.61 | 61 | 12.63 | -40.3 | 3.17 | 0.714 |
| 0.62 | 62 | 12.78 | -36.4 | 3.32 | 0.748 |
| 0.63 | 63 | 10.13 | -102.1 | 0.67 | 0.151 |
| 0.64 | 64 | 10.18 | -100.8 | 0.72 | 0.162 |
| 0.649 | 65 | 10.54 | -92.1 | 1.08 | 0.243 |
| 0.66 | 66 | 10.58 | -91.1 | 1.12 | 0.252 |
| 0.668 | 67 | 11.06 | -79 | 1.6 | 0.360 |
| 0.68 | 68 | 11.57 | -66.3 | 2.11 | 0.475 |
| 0.69 | 69 | 13.24 | -25 | 3.78 | 0.851 |
| 0.7 | 70 | 12.99 | -31.3 | 3.53 | 0.795 |
| 0.712 | 71 | 11.38 | -71.1 | 1.92 | 0.432 |
| 0.72 | 72 | 10.79 | -85.8 | 1.33 | 0.300 |
| 0.73 | 73 | 11.56 | -66.8 | 2.1 | 0.473 |
| 0.74 | 74 | 11.62 | -65.2 | 2.16 | 0.486 |
| 0.7513 | 75 | 10.99 | -80.9 | 1.53 | 0.345 |
| 0.76 | 76 | 10.93 | -82.3 | 1.47 | 0.331 |
| 0.769 | 77 | 11.96 | -56.7 | 2.5 | 0.563 |
| 0.78 | 78 | 13.31 | -23.4 | 3.85 | 0.867 |
| 0.79 | 79 | 11.6 | -65.8 | 2.14 | 0.482 |
| 0.8 | 80 | 11.06 | -79.1 | 1.6 | 0.360 |
| 0.811 | 81 | 12.35 | -47.1 | 2.89 | 0.651 |
| 0.82 | 82 | 12.43 | -45.2 | 2.97 | 0.669 |
| 0.832 | 83 | 12.41 | -45.7 | 2.95 | 0.664 |
| 0.84 | 84 | 12.9 | -33.5 | 3.44 | 0.775 |
| 0.851 | 85 | 13.26 | -24.6 | 3.8 | 0.856 |
| 0.86 | 86 | 13.14 | -27.5 | 3.68 | 0.829 |
| 0.872 | 87 | 10.96 | -81.4 | 1.5 | 0.338 |
| 0.88 | 88 | 10.84 | -84.7 | 1.38 | 0.311 |
| 0.89 | 89 | 11.22 | -75.2 | 1.76 | 0.396 |
| 0.9 | 90 | 11.84 | -59.8 | 2.38 | 0.536 |
| 0.911 | 91 | 12.2 | -50.8 | 2.74 | 0.617 |
| 0.92 | 92 | 11.99 | -56 | 2.53 | 0.570 |
| 0.929 | 93 | 12.26 | -49.3 | 2.8 | 0.631 |
| 0.94 | 94 | 13.18 | -26.6 | 3.72 | 0.838 |
| 0.95 | 95 | 13.95 | -7.4 | 4.49 | 1.011 |
| 0.96 | 96 | 12.03 | -55 | 2.57 | 0.579 |
| 0.9705 | 97 | 12 | -55.8 | 2.54 | 0.572 |
| 0.98 | 98 | 12.38 | -46.3 | 2.92 | 0.658 |
| 0.989 | 99 | 12.33 | -47.7 | 2.87 | 0.646 |
| 1 | 100 | 12.7 | -38.4 | 3.24 | 0.730 |
| 1.01 | 101 | 11.84 | -59.6 | 2.38 | 0.536 |
| 1.02 | 102 | 12.87 | -34.2 | 3.41 | 0.768 |
| 1.0299 | 103 | 12.95 | -32.2 | 3.49 | 0.786 |
| 1.04 | 104 | 11.04 | -79.6 | 1.58 | 0.356 |
| 1.0517 | 105 | 12.09 | -53.7 | 2.63 | 0.592 |
| 1.06 | 106 | 13.19 | -26.2 | 3.73 | 0.840 |
| 1.0694 | 107 | 14.63 | 2.9 | 5.17 | 1.164 |
| 1.08 | 108 | 13.06 | -29.5 | 3.6 | 0.811 |
| 1.092 | 109 | 12.51 | -43 | 3.05 | 0.687 |
| 1.1 | 110 | 12.38 | -46.4 | 2.92 | 0.658 |
| 1.1106 | 111 | 12.88 | -34 | 3.42 | 0.770 |
| 1.12 | 112 | 11.73 | -62.4 | 2.27 | 0.511 |
| 1.1293 | 113 | 10.96 | -81.4 | 1.5 | 0.338 |
| 1.14 | 114 | 12.13 | -52.5 | 2.67 | 0.601 |
| 1.15 | 115 | 12.67 | -39.1 | 3.21 | 0.723 |
| 1.16 | 116 | 13.24 | -25 | 3.78 | 0.851 |
| 1.1697 | 117 | 13.15 | -27.2 | 3.69 | 0.831 |
| 1.18 | 118 | 13.55 | -17.5 | 4.09 | 0.921 |
| 1.1899 | 119 | 12.65 | -39.6 | 3.19 | 0.718 |
| 1.2 | 120 | 11.55 | -66.9 | 2.09 | 0.471 |
| 1.211 | 121 | 10.96 | -81.6 | 1.5 | 0.338 |
| 1.2196 | 122 | 12.12 | -52.8 | 2.66 | 0.599 |
| 1.23 | 123 | 13.99 | -6.6 | 4.53 | 1.020 |
| 1.2399 | 124 | 14.16 | -2.3 | 4.7 | 1.059 |
| 1.2499 | 125 | 11.32 | -72.6 | 1.86 | 0.419 |
| 1.26 | 126 | 12.22 | -50.3 | 2.76 | 0.622 |
| 1.27 | 127 | 13.05 | -29.7 | 3.59 | 0.809 |
| 1.28 | 128 | 12.87 | -34.3 | 3.41 | 0.768 |
| 1.2891 | 129 | 11.47 | -68.9 | 2.01 | 0.453 |
| 1.3 | 130 | 12.16 | -51.9 | 2.7 | 0.608 |
| 1.3091 | 131 | 12.72 | -38 | 3.26 | 0.734 |
| 1.32 | 132 | 12.71 | -38.3 | 3.25 | 0.732 |
| 1.3301 | 133 | 12.04 | -54.8 | 2.58 | 0.581 |
| 1.34 | 134 | 12.29 | -48.6 | 2.83 | 0.637 |
| 1.3503 | 135 | 13.86 | -9.8 | 4.4 | 0.991 |
| 1.36 | 136 | 13.38 | -21.5 | 3.92 | 0.883 |
| 1.3699 | 137 | 11.54 | -67.2 | 2.08 | 0.468 |
| 1.38 | 138 | 12.19 | -51.2 | 2.73 | 0.615 |
| 1.39 | 139 | 13.71 | -13.4 | 4.25 | 0.957 |
| 1.4 | 140 | 13.73 | -12.9 | 4.27 | 0.962 |
| 1.4117 | 141 | 12.41 | -45.5 | 2.95 | 0.664 |
| 1.42 | 142 | 12.11 | -53.1 | 2.65 | 0.597 |
| 1.43 | 143 | 13.24 | -25 | 3.78 | 0.851 |
| 1.44 | 144 | 13.81 | -10.9 | 4.35 | 0.980 |
| 1.451 | 145 | 13.87 | -9.3 | 4.41 | 0.993 |
| 1.46 | 146 | 11.49 | -68.4 | 2.03 | 0.457 |
| 1.469 | 147 | 12.26 | -49.3 | 2.8 | 0.631 |
| 1.48 | 148 | 13.56 | -17 | 4.1 | 0.923 |
| 1.488 | 149 | 13.94 | -7.6 | 4.48 | 1.009 |
| 1.5 | 150 | 11.35 | -71.8 | 1.89 | 0.426 |
| 1.5088 | 151 | 11.44 | -69.7 | 1.98 | 0.446 |
| 1.52 | 152 | 12.64 | -39.8 | 3.18 | 0.716 |
| 1.532 | 153 | 11.72 | -62.7 | 2.26 | 0.509 |
| 1.5397 | 154 | 11.46 | -69.1 | 2 | 0.450 |
| 1.549 | 155 | 12.03 | -55 | 2.57 | 0.579 |
| 1.56 | 156 | 13.52 | -18 | 4.06 | 0.914 |
| 1.571 | 157 | 12.47 | -44.1 | 3.01 | 0.678 |
| 1.5796 | 158 | 11.63 | -65 | 2.17 | 0.489 |
| 1.5895 | 159 | 12.21 | -50.6 | 2.75 | 0.619 |
| 1.5995 | 160 | 13.01 | -30.7 | 3.55 | 0.800 |
| 1.612 | 161 | 13.43 | -20.4 | 3.97 | 0.894 |
| 1.62 | 162 | 12.81 | -35.6 | 3.35 | 0.755 |
| 1.6293 | 163 | 12.41 | -45.7 | 2.95 | 0.664 |
| 1.64 | 164 | 13.35 | -22.3 | 3.89 | 0.876 |
| 1.648 | 165 | 12.07 | -54.1 | 2.61 | 0.588 |
| 1.66 | 166 | 11.59 | -66 | 2.13 | 0.480 |
| 1.6704 | 167 | 12.11 | -53 | 2.65 | 0.597 |
| 1.68 | 168 | 13.14 | -27.6 | 3.68 | 0.829 |
| 1.69 | 169 | 12.98 | -31.4 | 3.52 | 0.793 |
| 1.7 | 170 | 11.62 | -65.3 | 2.16 | 0.486 |
| 1.709 | 171 | 12.75 | -37.2 | 3.29 | 0.741 |
| 1.7199 | 172 | 12.61 | -40.7 | 3.15 | 0.709 |
| 1.7294 | 173 | 12.77 | -36.7 | 3.31 | 0.745 |
| 1.74 | 174 | 12.83 | -35.2 | 3.37 | 0.759 |
| 1.7501 | 175 | 12.81 | -35.8 | 3.35 | 0.755 |
| 1.76 | 176 | 13.63 | -15.3 | 4.17 | 0.939 |
| 1.77 | 177 | 13.69 | -14 | 4.23 | 0.953 |
| 1.78 | 178 | 13.02 | -30.4 | 3.56 | 0.802 |
| 1.789 | 179 | 11.93 | -57.6 | 2.47 | 0.556 |
| 1.8 | 180 | 12.87 | -34.3 | 3.41 | 0.768 |
| 1.81 | 181 | 13.27 | -24.3 | 3.81 | 0.858 |
| 1.82 | 182 | 12.66 | -39.4 | 3.2 | 0.721 |
| 1.8304 | 183 | 13.16 | -29.5 | 3.7 | 0.833 |
| 1.84 | 184 | 12.08 | -50.9 | 2.62 | 0.590 |
| 1.85 | 185 | 13.12 | -30.4 | 3.66 | 0.824 |
| 1.86 | 186 | 13.1 | -30.7 | 3.64 | 0.820 |
| 1.87 | 187 | 13.58 | -21.2 | 4.12 | 0.928 |
| 1.88 | 188 | 12.78 | -37.1 | 3.32 | 0.748 |
| 1.89 | 189 | 13.33 | -26.2 | 3.87 | 0.872 |
| 1.9 | 190 | 13.63 | -20.2 | 4.17 | 0.939 |
| 1.91 | 191 | 12.46 | -43.5 | 3 | 0.676 |
| 1.92 | 192 | 12.06 | -51.3 | 2.6 | 0.586 |
| 1.931 | 193 | 13.29 | -26.9 | 3.83 | 0.863 |
| 1.94 | 194 | 13.25 | -27.8 | 3.79 | 0.854 |
| 1.9487 | 195 | 12.94 | -33.9 | 3.48 | 0.784 |
| 1.96 | 196 | 12.93 | -34.2 | 3.47 | 0.782 |
| 1.969 | 197 | 13.31 | -26.6 | 3.85 | 0.867 |
| 1.9795 | 198 | 13.71 | -18.6 | 4.25 | 0.957 |
| 1.99 | 199 | 12.78 | -37.1 | 3.32 | 0.748 |
| 2 | 200 | 12.82 | -36.4 | 3.36 | 0.757 |
| 2.01 | 201 | 13.45 | -23.7 | 3.99 | 0.899 |
| 2.02 | 202 | 13.54 | -22 | 4.08 | 0.919 |
| 2.03 | 203 | 13.39 | -25 | 3.93 | 0.885 |
| 2.04 | 204 | 12.85 | -35.6 | 3.39 | 0.764 |
| 2.05 | 205 | 12.98 | -33.2 | 3.52 | 0.793 |
| 2.0597 | 206 | 12.85 | -35.6 | 3.39 | 0.764 |
| 2.07 | 207 | 12.18 | -49 | 2.72 | 0.613 |
| 2.08 | 208 | 11.71 | -58.3 | 2.25 | 0.507 |
| 2.089 | 209 | 12.24 | -47.8 | 2.78 | 0.626 |
| 2.0997 | 210 | 13.84 | -16 | 4.38 | 0.986 |
| 2.111 | 211 | 13.06 | -31.6 | 3.6 | 0.811 |
| 2.12 | 212 | 13.62 | -20.5 | 4.16 | 0.937 |
| 2.129 | 213 | 12.65 | -39.7 | 3.19 | 0.718 |
| 2.1395 | 214 | 13.61 | -20.6 | 4.15 | 0.935 |
| 2.1495 | 215 | 11.72 | -58.2 | 2.26 | 0.509 |
| 2.16 | 216 | 12.55 | -41.6 | 3.09 | 0.696 |
| 2.17 | 217 | 13.17 | -29.4 | 3.71 | 0.836 |
| 2.18 | 218 | 13.21 | -28.5 | 3.75 | 0.845 |
| 2.189 | 219 | 13.61 | -20.6 | 4.15 | 0.935 |
| 2.2 | 220 | 13.12 | -30.4 | 3.66 | 0.824 |
| 2.21 | 221 | 13.6 | -20.8 | 4.14 | 0.932 |
| 2.22 | 222 | 13.4 | -24.7 | 3.94 | 0.887 |
| 2.23 | 223 | 14.09 | -11.1 | 4.63 | 1.043 |
| 2.24 | 224 | 13.37 | -25.5 | 3.91 | 0.881 |
| 2.25 | 225 | 12.98 | -33.2 | 3.52 | 0.793 |
| 2.26 | 226 | 14.13 | -10.3 | 4.67 | 1.052 |
| 2.2695 | 227 | 14.32 | -6.6 | 4.86 | 1.095 |
| 2.28 | 228 | 13.11 | -30.5 | 3.65 | 0.822 |
| 2.289 | 229 | 13.05 | -31.7 | 3.59 | 0.809 |
| 2.3 | 230 | 14.2 | -8.9 | 4.74 | 1.068 |
| 2.311 | 231 | 13.62 | -20.4 | 4.16 | 0.937 |
| 2.32 | 232 | 13.66 | -19.6 | 4.2 | 0.946 |
| 2.33 | 233 | 13.43 | -24.1 | 3.97 | 0.894 |
| 2.34 | 234 | 13.33 | -26.2 | 3.87 | 0.872 |
| 2.3512 | 235 | 14.69 | 0.2 | 5.23 | 1.178 |
| 2.36 | 236 | 12.93 | -34.2 | 3.47 | 0.782 |
| 2.369 | 237 | 12.63 | -40.2 | 3.17 | 0.714 |
| 2.38 | 238 | 13.65 | -19.8 | 4.19 | 0.944 |
| 2.391 | 239 | 14.92 | 1.6 | 5.46 | 1.230 |
| 2.4 | 240 | 14.14 | -10 | 4.68 | 1.054 |
| 2.411 | 241 | 12.88 | -35.2 | 3.42 | 0.770 |
| 2.42 | 242 | 13.24 | -27.9 | 3.78 | 0.851 |
| 2.43 | 243 | 13.78 | -17.2 | 4.32 | 0.973 |
| 2.44 | 244 | 13.07 | -31.3 | 3.61 | 0.813 |
| 2.45 | 245 | 12 | -52.7 | 2.54 | 0.572 |
| 2.4596 | 246 | 12.9 | -34.8 | 3.44 | 0.775 |
| 2.47 | 247 | 14.38 | -5.4 | 4.92 | 1.108 |
| 2.48 | 248 | 12.66 | -39.4 | 3.2 | 0.721 |
| 2.49 | 249 | 12.29 | -46.8 | 2.83 | 0.637 |
| 2.5 | 250 | 12.85 | -35.6 | 3.39 | 0.764 |
| 2.5092 | 251 | 12.67 | -39.3 | 3.21 | 0.723 |
| 2.52 | 252 | 12.39 | -44.8 | 2.93 | 0.660 |
| 2.53 | 253 | 12.45 | -43.6 | 2.99 | 0.673 |
| 2.54 | 254 | 13.63 | -20.2 | 4.17 | 0.939 |
| 2.5495 | 255 | 14.13 | -10.3 | 4.67 | 1.052 |
| 2.56 | 256 | 14.14 | -10 | 4.68 | 1.054 |
| 2.571 | 257 | 14.19 | -9.2 | 4.73 | 1.065 |
| 2.58 | 258 | 14.1 | -10.9 | 4.64 | 1.045 |
| 2.5893 | 259 | 14.04 | -12.1 | 4.58 | 1.032 |
| 2.6 | 260 | 13.75 | -17.9 | 4.29 | 0.966 |
| 2.6098 | 261 | 12.88 | -35.2 | 3.42 | 0.770 |
| 2.62 | 262 | 12.87 | -35.3 | 3.41 | 0.768 |
| 2.6293 | 263 | 15.25 | 3.6 | 5.79 | 1.304 |
| 2.64 | 264 | 14.64 | -0.3 | 5.18 | 1.167 |
| 2.65 | 265 | 12.68 | -39.1 | 3.22 | 0.725 |
| 2.6595 | 266 | 13.47 | -23.4 | 4.01 | 0.903 |
| 2.67 | 267 | 14.62 | -0.6 | 5.16 | 1.162 |
| 2.68 | 268 | 14.39 | -5.2 | 4.93 | 1.110 |
| 2.6902 | 269 | 12.74 | -37.8 | 3.28 | 0.739 |
| 2.7 | 270 | 12.9 | -34.8 | 3.44 | 0.775 |
| 2.7095 | 271 | 13.31 | -26.6 | 3.85 | 0.867 |
| 2.72 | 272 | 12.85 | -35.6 | 3.39 | 0.764 |
| 2.73 | 273 | 13.73 | -18.2 | 4.27 | 0.962 |
| 2.74 | 274 | 13.43 | -24.3 | 3.97 | 0.894 |
| 2.75 | 275 | 16.41 | 10.6 | 6.95 | 1.565 |
| 2.7599 | 276 | 15.06 | 2.5 | 5.6 | 1.261 |
| 2.77 | 277 | 14.55 | -1.9 | 5.09 | 1.146 |
| 2.78 | 278 | 14.01 | -12.6 | 4.55 | 1.025 |
| 2.789 | 279 | 14.58 | -1.3 | 5.12 | 1.153 |
| 2.8 | 280 | 14.33 | -6.3 | 4.87 | 1.097 |
| 2.81 | 281 | 13.76 | -17.8 | 4.3 | 0.968 |
| 2.82 | 282 | 12.74 | -38 | 3.28 | 0.739 |
| 2.8304 | 283 | 13.77 | -17.5 | 4.31 | 0.971 |
| 2.84 | 284 | 14.42 | -4.7 | 4.96 | 1.117 |
| 2.85 | 285 | 13.96 | -13.7 | 4.5 | 1.014 |
| 2.86 | 286 | 14.27 | -7.6 | 4.81 | 1.083 |
| 2.871 | 287 | 14.46 | -3.8 | 5 | 1.126 |
| 2.88 | 288 | 14.8 | 0.9 | 5.34 | 1.203 |
| 2.8904 | 289 | 13.94 | -14.1 | 4.48 | 1.009 |
| 2.9 | 290 | 14.17 | -9.6 | 4.71 | 1.061 |
| 2.9096 | 291 | 14.26 | -7.7 | 4.8 | 1.081 |
| 2.92 | 292 | 14.2 | -9 | 4.74 | 1.068 |
| 2.93 | 293 | 13.78 | -17.3 | 4.32 | 0.973 |
| 2.94 | 294 | 13.79 | -17 | 4.33 | 0.975 |
| 2.951 | 295 | 15.96 | 7.8 | 6.5 | 1.464 |
| 2.96 | 296 | 14.41 | -4.8 | 4.95 | 1.115 |
| 2.971 | 297 | 14.39 | -5.1 | 4.93 | 1.110 |
| 2.98 | 298 | 14.28 | -7.3 | 4.82 | 1.086 |
| 2.99 | 299 | 15.3 | 3.9 | 5.84 | 1.315 |
| 3 | 300 | 14.62 | -0.6 | 5.16 | 1.162 |
| 3.01 | 301 | 13.98 | -13.4 | 4.52 | 1.018 |
| 3.02 | 302 | 14.17 | -9.6 | 4.71 | 1.061 |
| 3.03 | 303 | 14.54 | -2.2 | 5.08 | 1.144 |
| 3.04 | 304 | 15.32 | 4 | 5.86 | 1.320 |
| 3.051 | 305 | 14.88 | 1.4 | 5.42 | 1.221 |
| 3.06 | 306 | 14.6 | -1 | 5.14 | 1.158 |
| 3.07 | 307 | 16.37 | 10.3 | 6.91 | 1.556 |
| 3.08 | 308 | 16.02 | 8.2 | 6.56 | 1.477 |
| 3.089 | 309 | 15.2 | 3.3 | 5.74 | 1.293 |
| 3.1 | 310 | 15.16 | 3 | 5.7 | 1.284 |
| 3.11 | 311 | 15.1 | 2.7 | 5.64 | 1.270 |
| 3.12 | 312 | 14.33 | -6.3 | 4.87 | 1.097 |
| 3.1299 | 313 | 14.58 | -1.3 | 5.12 | 1.153 |
| 3.14 | 314 | 13.74 | -18 | 4.28 | 0.964 |
| 3.151 | 315 | 14.57 | -1.6 | 5.11 | 1.151 |
| 3.1596 | 316 | 16.01 | 8.2 | 6.55 | 1.475 |
| 3.171 | 317 | 15.7 | 6.3 | 6.24 | 1.405 |
| 3.18 | 318 | 15.01 | 2.2 | 5.55 | 1.250 |
| 3.189 | 319 | 15.85 | 7.2 | 6.39 | 1.439 |
| 3.2 | 320 | 16.22 | 9.4 | 6.76 | 1.523 |
| 3.2097 | 321 | 16.6 | 11.7 | 7.14 | 1.608 |
| 3.2198 | 322 | 16.4 | 10.5 | 6.94 | 1.563 |
| 3.2299 | 323 | 16.22 | 9.4 | 6.76 | 1.523 |
| 3.24 | 324 | 15.76 | 6.6 | 6.3 | 1.419 |
| 3.25 | 325 | 16.3 | 9.9 | 6.84 | 1.541 |
| 3.26 | 326 | 15.97 | 7.9 | 6.51 | 1.466 |
| 3.2714 | 327 | 15.37 | 4.3 | 5.91 | 1.331 |
| 3.28 | 328 | 14.53 | -2.3 | 5.07 | 1.142 |
| 3.29 | 329 | 17.16 | 15 | 7.7 | 1.734 |
| 3.3 | 330 | 14.06 | -11.6 | 4.6 | 1.036 |
| 3.3093 | 331 | 14.06 | -11.6 | 4.6 | 1.036 |
| 3.32 | 332 | 13.84 | -16.1 | 4.38 | 0.986 |
| 3.33 | 333 | 14.41 | -4.8 | 4.95 | 1.115 |
| 3.34 | 334 | 14.14 | -10.2 | 4.68 | 1.054 |
| 3.35 | 335 | 14.09 | -11.1 | 4.63 | 1.043 |
| 3.36 | 336 | 14.25 | -8 | 4.79 | 1.079 |
| 3.37 | 337 | 14.65 | 0 | 5.19 | 1.169 |
| 3.38 | 338 | 14.78 | 0.8 | 5.32 | 1.198 |
| 3.39 | 339 | 14.33 | -6.3 | 4.87 | 1.097 |
| 3.4 | 340 | 15.62 | 5.8 | 6.16 | 1.387 |
| 3.41 | 341 | 15.94 | 7.8 | 6.48 | 1.459 |
| 3.42 | 342 | 15.41 | 4.6 | 5.95 | 1.340 |
| 3.429 | 343 | 15.05 | 2.4 | 5.59 | 1.259 |
| 3.44 | 344 | 15.06 | 2.5 | 5.6 | 1.261 |
| 3.451 | 345 | 16.7 | 12.3 | 7.24 | 1.631 |
| 3.46 | 346 | 16.58 | 11.6 | 7.12 | 1.604 |
| 3.47 | 347 | 16.61 | 11.8 | 7.15 | 1.610 |
| 3.48 | 348 | 16.61 | 11.8 | 7.15 | 1.610 |
| 3.49 | 349 | 16.34 | 10.2 | 6.88 | 1.550 |
| 3.5 | 350 | 16.68 | 12.2 | 7.22 | 1.626 |
| 3.51 | 351 | 16.44 | 10.7 | 6.98 | 1.572 |
| 3.52 | 352 | 15.86 | 7.3 | 6.4 | 1.441 |
| 3.53 | 353 | 16.53 | 11.3 | 7.07 | 1.592 |
| 3.54 | 354 | 16.66 | 12.1 | 7.2 | 1.622 |
| 3.549 | 355 | 15.22 | 3.4 | 5.76 | 1.297 |
| 3.56 | 356 | 15.61 | 5.8 | 6.15 | 1.385 |
| 3.57 | 357 | 15.78 | 6.8 | 6.32 | 1.423 |
| 3.58 | 358 | 15.65 | 6 | 6.19 | 1.394 |
| 3.59 | 359 | 15.46 | 4.9 | 6 | 1.351 |
| 3.6 | 360 | 15.49 | 5 | 6.03 | 1.358 |
| 3.61 | 361 | 16.25 | 9.6 | 6.79 | 1.529 |
| 3.62 | 362 | 16.25 | 9.6 | 6.79 | 1.529 |
| 3.631 | 363 | 14.27 | -7.6 | 4.81 | 1.083 |
| 3.64 | 364 | 14.55 | -2 | 5.09 | 1.146 |
| 3.65 | 365 | 16.21 | 9.4 | 6.75 | 1.520 |
| 3.66 | 366 | 16.13 | 8.9 | 6.67 | 1.502 |
| 3.669 | 367 | 14.03 | -12.2 | 4.57 | 1.029 |
| 3.68 | 368 | 14.3 | -7 | 4.84 | 1.090 |
| 3.69 | 369 | 15.85 | 7.2 | 6.39 | 1.439 |
| 3.7 | 370 | 16.89 | 13.4 | 7.43 | 1.673 |
| 3.711 | 371 | 16.41 | 10.6 | 6.95 | 1.565 |
| 3.72 | 372 | 14.85 | 1.2 | 5.39 | 1.214 |
| 3.731 | 373 | 16.69 | 12.2 | 7.23 | 1.628 |
| 3.74 | 374 | 16.76 | 12.6 | 7.3 | 1.644 |
| 3.749 | 375 | 16.6 | 11.7 | 7.14 | 1.608 |
| 3.76 | 376 | 14.86 | 1.3 | 5.4 | 1.216 |
| 3.77 | 377 | 15.76 | 6.6 | 6.3 | 1.419 |
| 3.78 | 378 | 17.76 | 18.6 | 8.3 | 1.869 |
| 3.79 | 379 | 15.76 | 6.6 | 6.3 | 1.419 |
| 3.8 | 380 | 16.05 | 8.4 | 6.59 | 1.484 |
| 3.811 | 381 | 15.8 | 6.9 | 6.34 | 1.428 |
| 3.82 | 382 | 15.92 | 7.6 | 6.46 | 1.455 |
| 3.829 | 383 | 15.94 | 7.8 | 6.48 | 1.459 |
| 3.84 | 384 | 15.44 | 4.7 | 5.98 | 1.347 |
| 3.85 | 385 | 15.7 | 6.3 | 6.24 | 1.405 |
| 3.86 | 386 | 17.73 | 18.5 | 8.27 | 1.863 |
| 3.872 | 387 | 17.5 | 17.1 | 8.04 | 1.811 |
| 3.88 | 388 | 15.41 | 4.6 | 5.95 | 1.340 |
| 3.891 | 389 | 16.14 | 9 | 6.68 | 1.505 |
| 3.9 | 390 | 16.53 | 11.3 | 7.07 | 1.592 |
| 3.912 | 391 | 16.4 | 10.5 | 6.94 | 1.563 |
| 3.92 | 392 | 16.86 | 13.3 | 7.4 | 1.667 |
| 3.931 | 393 | 15.68 | 6.2 | 6.22 | 1.401 |
| 3.94 | 394 | 17.45 | 16.8 | 7.99 | 1.800 |
| 3.95 | 395 | 15.94 | 7.8 | 6.48 | 1.459 |
| 3.96 | 396 | 15.81 | 7 | 6.35 | 1.430 |
| 3.97 | 397 | 15.04 | 2.3 | 5.58 | 1.257 |
| 3.98 | 398 | 14.85 | 1.2 | 5.39 | 1.214 |
| 3.99 | 399 | 14.59 | -1.2 | 5.13 | 1.155 |
| 4 | 400 | 14.17 | -9.6 | 4.71 | 1.061 |
| 4.011 | 401 | 15.58 | 5.6 | 6.12 | 1.378 |
| 4.02 | 402 | 15.82 | 7 | 6.36 | 1.432 |
| 4.03 | 403 | 16.42 | 10.6 | 6.96 | 1.568 |
| 4.04 | 404 | 15.76 | 6.6 | 6.3 | 1.419 |
| 4.049 | 405 | 14.58 | -1.3 | 5.12 | 1.153 |
| 4.06 | 406 | 15.6 | 5.7 | 6.14 | 1.383 |
| 4.069 | 407 | 15.66 | 6.1 | 6.2 | 1.396 |
| 4.08 | 408 | 16.1 | 8.7 | 6.64 | 1.495 |
| 4.089 | 409 | 15.33 | 4.1 | 5.87 | 1.322 |
| 4.1 | 410 | 16.22 | 9.4 | 6.76 | 1.523 |
| 4.111 | 411 | 16.74 | 12.6 | 7.28 | 1.640 |
| 4.12 | 412 | 16.3 | 9.9 | 6.84 | 1.541 |
| 4.13 | 413 | 16.66 | 12.1 | 7.2 | 1.622 |
| 4.14 | 414 | 16.5 | 11.1 | 7.04 | 1.586 |
| 4.149 | 415 | 15.22 | 3.4 | 5.76 | 1.297 |
| 4.16 | 416 | 15.98 | 8 | 6.52 | 1.468 |
| 4.169 | 417 | 16.18 | 9.2 | 6.72 | 1.514 |
| 4.18 | 418 | 16.76 | 12.6 | 7.3 | 1.644 |
| 4.19 | 419 | 16.61 | 11.8 | 7.15 | 1.610 |
| 4.2 | 420 | 16.64 | 11.9 | 7.18 | 1.617 |
| 4.21 | 421 | 16.18 | 9.2 | 6.72 | 1.514 |
| 4.22 | 422 | 17.01 | 14.2 | 7.55 | 1.700 |
| 4.229 | 423 | 15.73 | 6.5 | 6.27 | 1.412 |
| 4.24 | 424 | 16.06 | 8.5 | 6.6 | 1.486 |
| 4.249 | 425 | 16.85 | 13.2 | 7.39 | 1.664 |
| 4.26 | 426 | 16.06 | 8.5 | 6.6 | 1.486 |
| 4.27 | 427 | 16.36 | 10.2 | 6.9 | 1.554 |
| 4.2795 | 428 | 17.24 | 15.5 | 7.78 | 1.752 |
| 4.29 | 429 | 16.25 | 9.6 | 6.79 | 1.529 |
| 4.3 | 430 | 17.01 | 14.2 | 7.55 | 1.700 |
| 4.311 | 431 | 15.98 | 8 | 6.52 | 1.468 |
| 4.32 | 432 | 17.56 | 17.4 | 8.1 | 1.824 |
| 4.329 | 433 | 17.06 | 14.5 | 7.6 | 1.712 |
| 4.34 | 434 | 16.9 | 13.5 | 7.44 | 1.676 |
| 4.35 | 435 | 15.74 | 6.6 | 6.28 | 1.414 |
| 4.36 | 436 | 16.13 | 8.9 | 6.67 | 1.502 |
| 4.369 | 437 | 17.21 | 15.4 | 7.75 | 1.745 |
| 4.38 | 438 | 16.34 | 10.2 | 6.88 | 1.550 |
| 4.39 | 439 | 15.69 | 6.2 | 6.23 | 1.403 |
| 4.4 | 440 | 15.84 | 7.1 | 6.38 | 1.437 |
| 4.409 | 441 | 16.33 | 10.1 | 6.87 | 1.547 |
| 4.42 | 442 | 16.12 | 8.8 | 6.66 | 1.500 |
| 4.431 | 443 | 17.16 | 15 | 7.7 | 1.734 |
| 4.44 | 444 | 17.52 | 17.2 | 8.06 | 1.815 |
| 4.45 | 445 | 16.69 | 12.2 | 7.23 | 1.628 |
| 4.461 | 446 | 17.04 | 14.3 | 7.58 | 1.707 |
| 4.47 | 447 | 16.26 | 9.7 | 6.8 | 1.532 |
| 4.48 | 448 | 16.58 | 11.6 | 7.12 | 1.604 |
| 4.489 | 449 | 16.98 | 14 | 7.52 | 1.694 |
| 4.5 | 450 | 16.04 | 8.3 | 6.58 | 1.482 |
| 4.511 | 451 | 16.3 | 9.9 | 6.84 | 1.541 |
| 4.521 | 452 | 16.66 | 12.1 | 7.2 | 1.622 |
| 4.53 | 453 | 16.9 | 13.5 | 7.44 | 1.676 |
| 4.54 | 454 | 15.65 | 6 | 6.19 | 1.394 |
| 4.551 | 455 | 15.48 | 5 | 6.02 | 1.356 |
| 4.56 | 456 | 15.12 | 2.8 | 5.66 | 1.275 |
| 4.57 | 457 | 15.76 | 6.6 | 6.3 | 1.419 |
| 4.58 | 458 | 16.06 | 8.5 | 6.6 | 1.486 |
| 4.589 | 459 | 15.72 | 6.4 | 6.26 | 1.410 |
| 4.6 | 460 | 15.17 | 3.1 | 5.71 | 1.286 |
| 4.609 | 461 | 15.38 | 4.4 | 5.92 | 1.333 |
| 4.62 | 462 | 16.32 | 10 | 6.86 | 1.545 |
| 4.631 | 463 | 15.78 | 6.8 | 6.32 | 1.423 |
| 4.64 | 464 | 16.09 | 8.6 | 6.63 | 1.493 |
| 4.651 | 465 | 15.77 | 6.7 | 6.31 | 1.421 |
| 4.66 | 466 | 16.58 | 11.6 | 7.12 | 1.604 |
| 4.67 | 467 | 14.45 | -3.9 | 4.99 | 1.124 |
| 4.68 | 468 | 15.16 | 3 | 5.7 | 1.284 |
| 4.69 | 469 | 15.65 | 6 | 6.19 | 1.394 |
| 4.7 | 470 | 15.94 | 7.8 | 6.48 | 1.459 |
| 4.71 | 471 | 14.45 | -3.9 | 4.99 | 1.124 |
| 4.72 | 472 | 14.53 | -2.5 | 5.07 | 1.142 |
| 4.73 | 473 | 16.4 | 10.5 | 6.94 | 1.563 |
| 4.74 | 474 | 16.93 | 13.7 | 7.47 | 1.682 |
| 4.751 | 475 | 15 | 2.1 | 5.54 | 1.248 |
| 4.76 | 476 | 14.78 | 0.8 | 5.32 | 1.198 |
| 4.771 | 477 | 16.38 | 10.4 | 6.92 | 1.559 |
| 4.78 | 478 | 15.84 | 7.1 | 6.38 | 1.437 |
| 4.79 | 479 | 14.58 | -1.5 | 5.12 | 1.153 |
| 4.8 | 480 | 16.2 | 9.3 | 6.74 | 1.518 |
| 4.81 | 481 | 16.82 | 13 | 7.36 | 1.658 |
| 4.82 | 482 | 16.78 | 12.8 | 7.32 | 1.649 |
| 4.83 | 483 | 14.5 | -3 | 5.04 | 1.135 |
| 4.84 | 484 | 14.94 | 1.8 | 5.48 | 1.234 |
| 4.85 | 485 | 16.72 | 12.4 | 7.26 | 1.635 |
| 4.86 | 486 | 15.77 | 6.7 | 6.31 | 1.421 |
| 4.87 | 487 | 14.2 | -9 | 4.74 | 1.068 |
| 4.88 | 488 | 14.17 | -9.6 | 4.71 | 1.061 |
| 4.89 | 489 | 15.17 | 3.1 | 5.71 | 1.286 |
| 4.9 | 490 | 16.29 | 9.8 | 6.83 | 1.538 |
| 4.911 | 491 | 16.25 | 9.6 | 6.79 | 1.529 |
| 4.92 | 492 | 16.4 | 10.5 | 6.94 | 1.563 |
| 4.93 | 493 | 15.1 | 2.7 | 5.64 | 1.270 |
| 4.94 | 494 | 15.54 | 5.4 | 6.08 | 1.369 |
| 4.95 | 495 | 16.33 | 10.1 | 6.87 | 1.547 |
| 4.96 | 496 | 17.08 | 14.6 | 7.62 | 1.716 |
| 4.969 | 497 | 19.08 | 26.6 | 9.62 | 2.167 |
| 4.98 | 498 | 16.96 | 13.8 | 7.5 | 1.689 |
| 4.988 | 499 | 16.84 | 13.1 | 7.38 | 1.662 |
| 5 | 500 | 18.32 | 22 | 8.86 | 1.995 |
| 5.01 | 501 | 16.45 | 10.8 | 6.99 | 1.574 |
| 5.02 | 502 | 16.88 | 13.4 | 7.42 | 1.671 |
| 5.029 | 503 | 17.14 | 15 | 7.68 | 1.730 |
| 5.04 | 504 | 17.58 | 17.6 | 8.12 | 1.829 |
| 5.049 | 505 | 16.65 | 12 | 7.19 | 1.619 |
| 5.061 | 506 | 16.26 | 9.7 | 6.8 | 1.532 |
| 5.07 | 507 | 16.82 | 13 | 7.36 | 1.658 |
| 5.082 | 508 | 17.82 | 19 | 8.36 | 1.883 |
| 5.091 | 509 | 17.4 | 16.5 | 7.94 | 1.788 |
| 5.102 | 510 | 16.61 | 11.8 | 7.15 | 1.610 |
| 5.111 | 511 | 16.09 | 8.6 | 6.63 | 1.493 |
| 5.12 | 512 | 16.74 | 12.6 | 7.28 | 1.640 |
| 5.13 | 513 | 17.84 | 19.1 | 8.38 | 1.887 |
| 5.14 | 514 | 17.33 | 16.1 | 7.87 | 1.773 |
| 5.15 | 515 | 17.5 | 17.1 | 8.04 | 1.811 |
| 5.16 | 516 | 17.33 | 16.1 | 7.87 | 1.773 |
| 5.17 | 517 | 17.37 | 16.3 | 7.91 | 1.782 |
| 5.179 | 518 | 18.18 | 21.2 | 8.72 | 1.964 |
| 5.19 | 519 | 16.98 | 14 | 7.52 | 1.694 |
| 5.2 | 520 | 17.97 | 19.9 | 8.51 | 1.917 |
| 5.211 | 521 | 17.78 | 18.8 | 8.32 | 1.874 |
| 5.22 | 522 | 17.25 | 15.6 | 7.79 | 1.755 |
| 5.232 | 523 | 17.94 | 19.8 | 8.48 | 1.910 |
| 5.24 | 524 | 17.6 | 17.7 | 8.14 | 1.833 |
| 5.249 | 525 | 17.8 | 18.9 | 8.34 | 1.878 |
| 5.261 | 526 | 17.9 | 19.5 | 8.44 | 1.901 |
| 5.271 | 527 | 17.06 | 14.5 | 7.6 | 1.712 |
| 5.281 | 528 | 17.49 | 17 | 8.03 | 1.809 |
| 5.29 | 529 | 17.65 | 18 | 8.19 | 1.845 |
| 5.3 | 530 | 16.52 | 11.2 | 7.06 | 1.590 |
| 5.309 | 531 | 17.77 | 18.7 | 8.31 | 1.872 |
| 5.32 | 532 | 17.62 | 17.8 | 8.16 | 1.838 |
| 5.33 | 533 | 18.6 | 23.7 | 9.14 | 2.059 |
| 5.34 | 534 | 17.12 | 14.6 | 7.66 | 1.725 |
| 5.35 | 535 | 17.31 | 17.4 | 7.85 | 1.768 |
| 5.361 | 536 | 17.4 | 18.7 | 7.94 | 1.788 |
| 5.371 | 537 | 17.1 | 14.2 | 7.64 | 1.721 |
| 5.381 | 538 | 17.18 | 15.5 | 7.72 | 1.739 |
| 5.391 | 539 | 17.44 | 19.4 | 7.98 | 1.797 |
| 5.4 | 540 | 17.21 | 15.9 | 7.75 | 1.745 |

**Table S2 Description of the input data used for the generation of the Oscillayers and their sources.**

| **Input data** | **Description** | **File type** | **Source** |
| --- | --- | --- | --- |
| Bioclim variables (present) | Bioclim variables for current conditions | ASCII (.asc) | http://worldclim.org/current |
| Bioclim variables (LGM) | Bioclim variables for conditions of the LGM (CCSM4) | ASCII (.asc) | [http://www.worldclim.org/palaeo-climate1](http://www.worldclim.org/paleo-climate1) |
| Delta layers | Interpolated differences between current and LGM bioclim variables | ASCII (.asc) | present study |
| Scaling factors | Factor used to scale the delta layers. Derived from the isotope record (Hansen et al., 2013) | Text (.csv) | present study |
| Palaeo-coastlines | ETOPO1 Global Relief Model reclassified corresponding changes in eustatic sea level (Hansen et al., 2013) | Shapefile (.shp) | present study |

**Supplementary Figures**

**
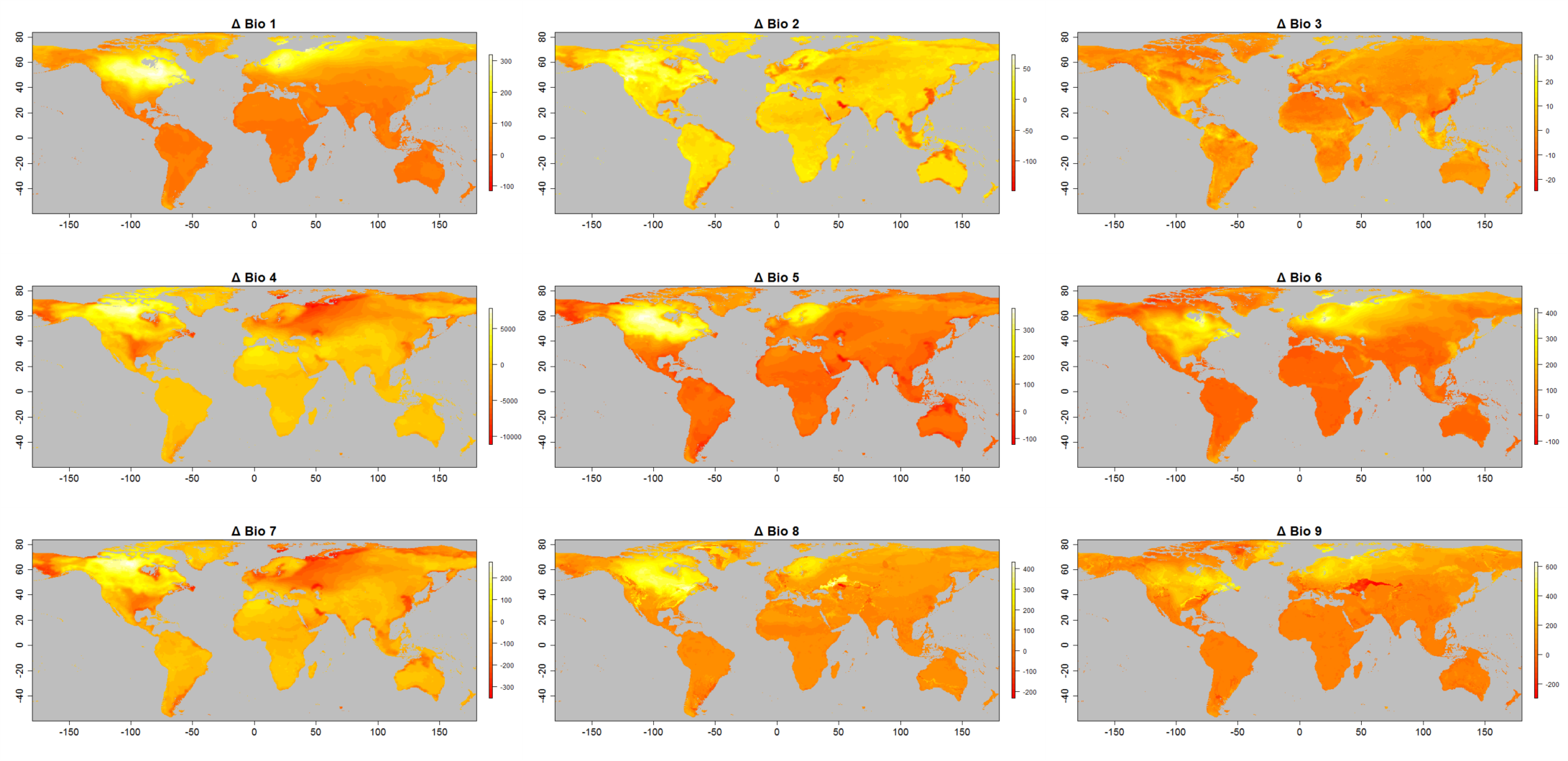
**

**FIGURE S1 Δ layers between the** **present (ISP) and the LGM (ISL) for the 19 bioclim variables.** Differences are in the units of the respective bioclim variable.

**
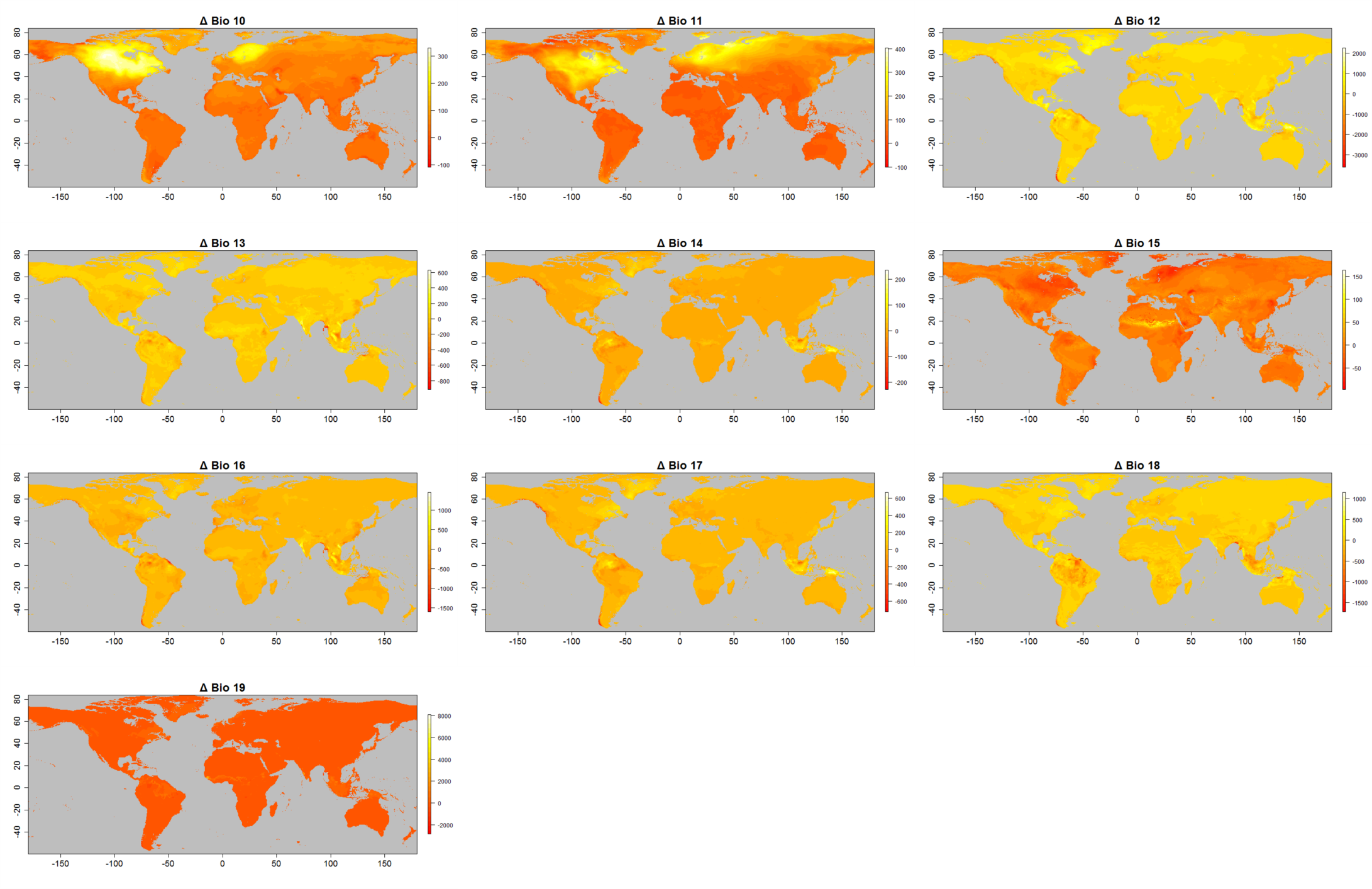
FIGURE S1** Continued.

**
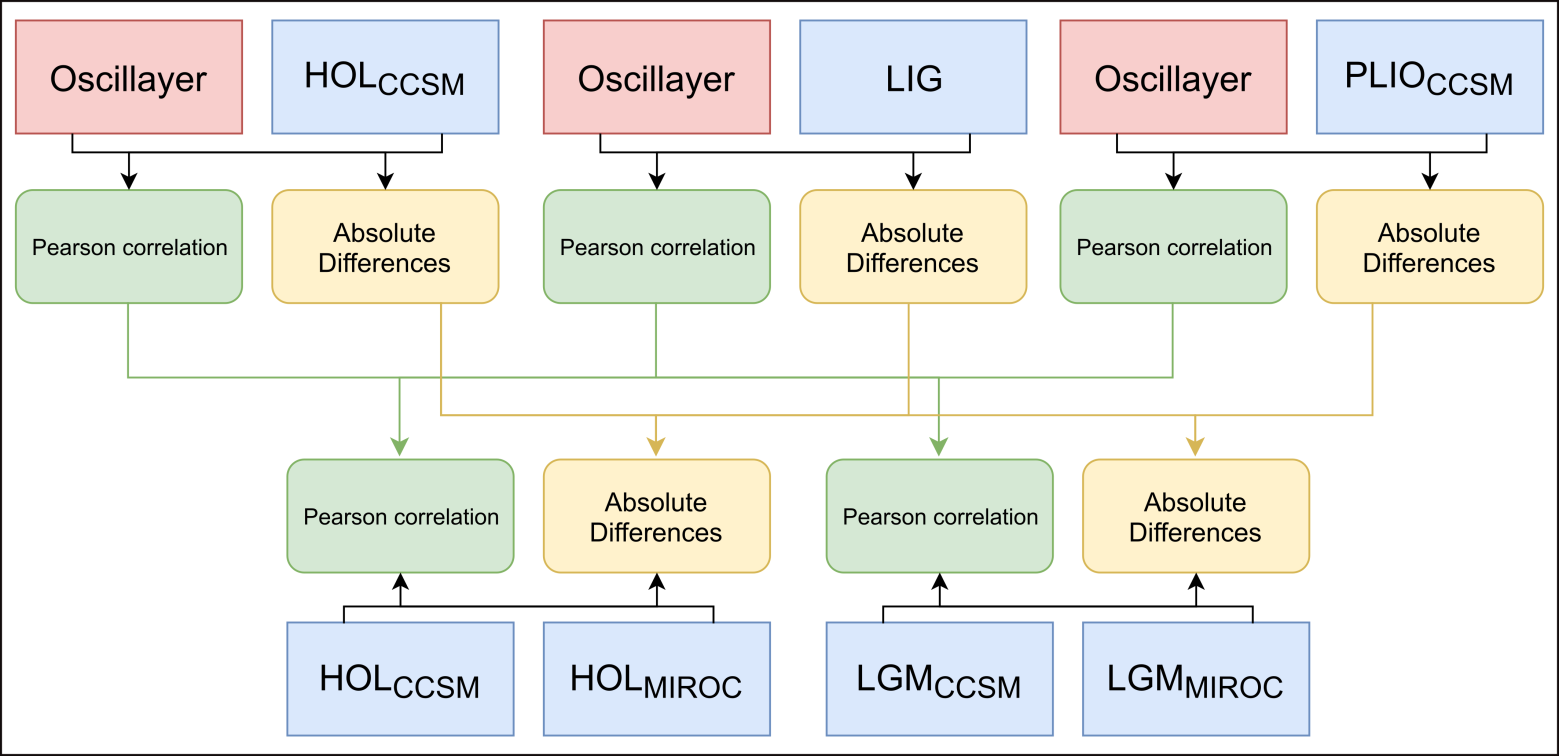
**

**FIGURE S2 Visualisation of the validation procedure.** Oscillayers for a given time period (red) were compared to respective modelled past climates (blue, e.g. HOL_CCSM_) in terms of Pearson correlations and absolute differences. The results of those latter comparisons (e.g. Oscillayer-HOL_CCSM_) were then evaluated against inter-model correlations (green arrows) and absolute differences (yellow arrows) for the HOL and LGM (e.g. HOL_CCSM_-HOL_MIROC_), respectively (see also Tab. 1).


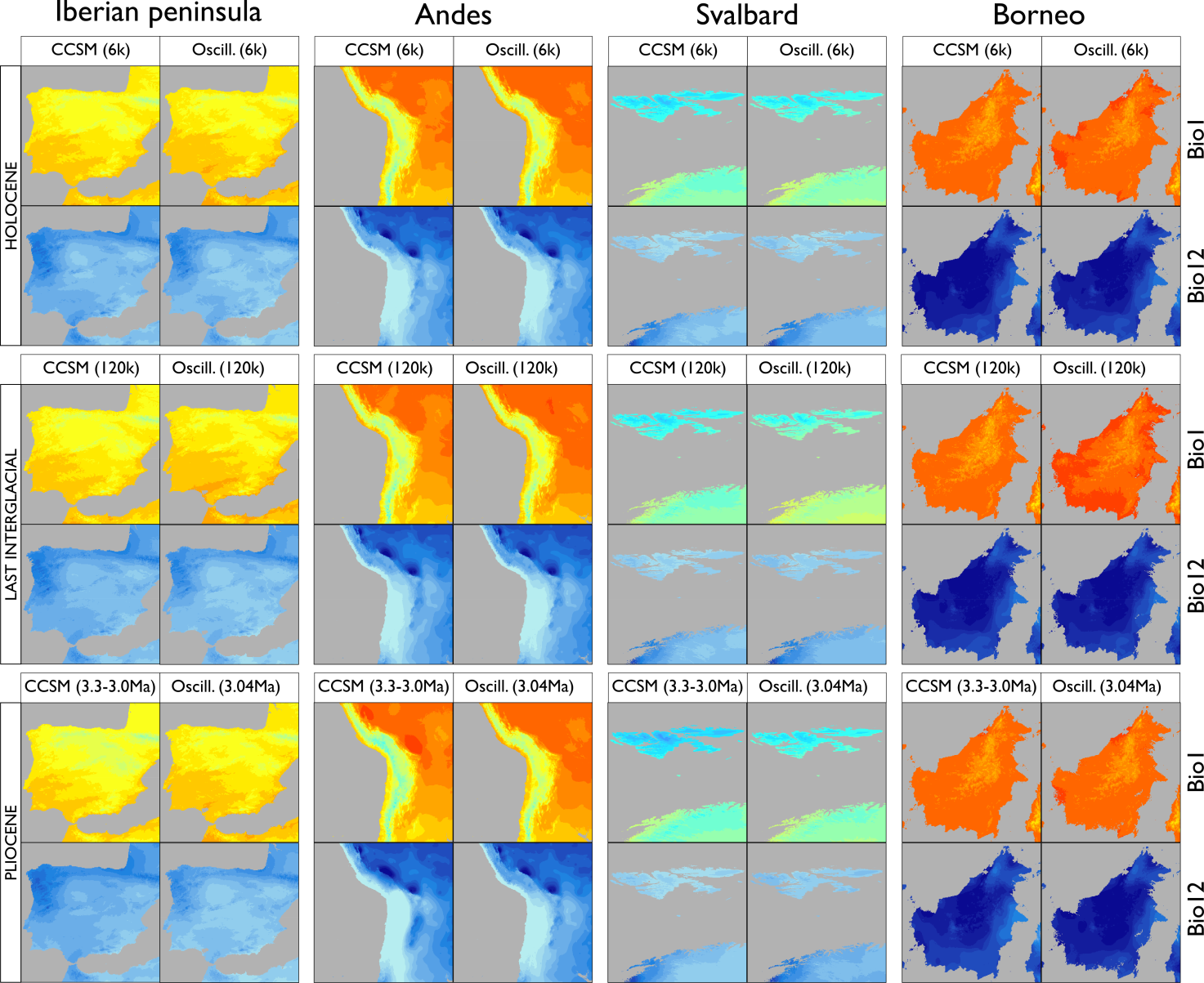


**FIGURE S3** Comparison of GCM-derived and interpolated layers (Oscillayers) for Annual Mean Temperature (Bio1) and Annual Precipitation (Bio12) in additional regions (Western Europe, Andes, Svalbard and Borneo). See Figure 1 for legend.
